# Evaluating the Efficacy of Pre-incisional Infiltration and Intraperitoneal Instillation of a Local Anesthetic Agent on Postoperative Analgesia and Hemodynamics in Patients Undergoing Laparoscopic Cholecystectomy Under General Anesthesia

**DOI:** 10.7759/cureus.22977

**Published:** 2022-03-08

**Authors:** Prashant K Mishra, Shreya Mani, Rakesh Bahadur Singh

**Affiliations:** 1 Anaesthesiology, G.S. Medical College & Hospital, Hapur, IND; 2 Anaesthesiology, Institute of Postgraduate Medical Education and Research, Kolkata, IND; 3 Anaesthesiology, Uttar Pradesh University of Medical Sciences (UPUMS), Etawah, IND

**Keywords:** hemodynamics, local anesthetic agent, pre-incisional infiltration, lap cholecystectomy, intra-peritoneal instillation, haemodynamics

## Abstract

Aim

The study aimed to compare the effects of 0.5% bupivacaine injection at pre-incisional port sites and intraperitoneal application on postoperative pain in laparoscopic cholecystectomy cases.

Methods

After taking ethical clearance, a total of 60 patients of the American Society of Anaesthesia (ASA) grades 1 and 2 scheduled to undergo laparoscopic cholecystectomy were enrolled in the study and were randomized into two groups. Group 1 (n=30) received 20 ml of 0.5% bupivacaine infiltration subcutaneously over the port sites before being given incision. Group 2 (n=30) received 20 ml of 0.5% bupivacaine applied in the intraperitoneal subdiaphragmatic space and in the gall bladder fossa after removal of the gall bladder. The efficacy in terms of abdominal pain, hemodynamics, complications, and total analgesic requirements were assessed at regular intervals throughout the postoperative period for 24 hours.

Results

No significant difference in terms of demographic variables in the groups. The mean visual analog score (VAS) score for abdominal pain was found to be significantly lower in group 1 from the first postoperative hour till the twenty-fourth hour. Also, no significant difference was seen between the groups regarding hemodynamic parameters. No significant difference between the groups was seen regarding postoperative nausea and vomiting (PONV). The supplemental analgesic requirement was significantly higher in group 2 than in group 1.

Conclusion

It was observed from this study that pre-incisional infiltration of a local anesthetic agent produces effective postoperative analgesia in the immediate postoperative hours and reduces additional analgesic requirements without causing any adverse reactions.

## Introduction

Since 1987, the standard procedure for the treatment of gall bladder stones is laparoscopic cholecystectomy (LC). The major benefit of using laparoscopy for upper gastrointestinal (GI) surgery is that it avoids an upper abdominal incision. Such upper abdominal incisions otherwise can hinder postoperative pulmonary rehabilitation, cause surgical wound pain, and increase the total medical cost [1-3]. Pain after LC is less intense and lasts a shorter time as compared to pain after open cholecystectomy [1-3]. Pain intensity usually peaks during the first postoperative period and usually declines over the following two to three days [4]. The postoperative pain seen after laparoscopic procedures has two components: visceral and somatic. The visceral component is due to the handling of tissues during the surgery, which causes tissue injury and the stretching of nerve endings [5]. The somatic component of pain is due to the incision made in the abdomen for the entry of the trocar [6]. Postoperative pain management is a major challenge after laparoscopic surgeries [7]. Various methods have been introduced to control postoperative pain after LC such as the use of local anesthetics (LAs), intraperitoneal infiltration of LA, preoperative administration of non-steroidal anti-inflammatory drugs (NSAIDs), combined use of NSAIDs and opioids, etc. The intraperitoneal instillation of LAs has been proposed to minimize postoperative pain after laparoscopic surgery [8]. The variable analgesic effects of periportal infiltration of local anesthetics, intraperitoneal spraying above the gall bladder, and instillation into the sub-diaphragmatic space and into the subhepatic space covering the area of the hepato-duodenal ligament have been reported [9]. Despite some studies, the role of local anesthetic agents during laparoscopic cholecystectomy is still not established. It is an exciting proposition that just the addition of a small maneuver during an already established operative procedure can decrease postoperative pain and morbidity. If its efficacy and safety can be proven it can become routine practice. There is no conclusive evidence to demonstrate which technique is superior for providing postoperative analgesia - pre-incisional local anesthetic infiltration or intraperitoneal local anesthetic instillation. Hence, we undertook this study to compare these two techniques.

## Materials and methods

The study was conducted after approval from the institute’s ethical committee. The proposed anesthetic technique was explained to the patients and written and informed consent was taken. A total of 60 patients were enrolled for the study and divided into two equal groups with 30 patients in each group. Group 1 (n=30) received pre-incisional infiltration with 20 ml of 0.5% bupivacaine. Group 2 (n=30) received 20 ml of 0.5% bupivacaine in the right subdiaphragmatic space and gallbladder fossa following the removal of the gall bladder. The inclusion and exclusion criteria listed in Table 1 were followed for the selection of patients for our study.

**Table 1:** Inclusion and exclusion criteria ASA: American Society of Anaesthesia; LA: local anesthesia; BMI: body mass index

Inclusion criteria	Exclusion criteria
ASA Physical Status 1 and 2	Patients refusal to give consent
Age 20-70 years, either sex	ASA grade 3 & 4
BMI 18-30 kg/m^2^	Patients with a cardiac, renal, or neurological disorder
All patients undergoing elective laparoscopic cholecystectomy	Patients with allergies to LA
--	Patients in whom the procedure was converted to open cholecystectomy, etc.

The patients were monitored for non-invasive blood pressure (NIBP), electrocardiograph (ECG), end-tidal CO2 (EtCO2), arterial oxygen saturation (SpO2), heart rate (HR), etc. The patients were premedicated with inj. glycopyrrolate (0.2 mg), inj. ondansetron (4 mg), and inj. tramadol (100 mg). Induction was done with 1.5-2 mg/kg propofol followed by 2 mg/kg succinylcholine for muscle relaxation and then a proper size of the endotracheal tube (ETT) was inserted. Just after ETT placement, inj. vecuronium 0.1 mg/kg was given. Anesthesia was maintained with isoflurane with 50% O2 and 50% N2O. Four trocars were introduced by a surgeon. A 10-mm trocar was placed in the infraumbilical region, a 10-mm trocar in the mid epigastrium 5 mm below the xiphoid, a 5-mm trocar in the right subcostal region in the midclavicular line, and a 5 mm trocar in the anterior axillary line. Intraabdominal pressure was maintained between 12 to 15 mmHg. For group 1 patients, before giving an incision for the ports, 20 ml of 0.5% bupivacaine was infiltrated subcutaneously over the port sites (6 ml was infiltrated around each midline port site and 4 ml was infiltrated around at the lateral port sites). For group 2 patients, after removal of the gall bladder before removing the trocar, 20 ml of 0.5% bupivacaine was instilled intraperitoneally over the subdiaphragmatic space and in the gall bladder bed with a separate catheter passed through one of the trocars. In the postanesthesia care unit (PACU) patients were asked about their intensity of pain according to the visual analog scale (VAS) in the resting condition. VAS score, postoperative nausea vomiting, and postoperative vitals were recorded at 0, 1, 2, 4, 8, 12, and 24 hours. Extubation time was considered as 0 hours. Inj. diclofenac 75 mg IV BD was given for 24 hours for postoperative analgesia. Additionally, inj. tramadol 100 mg IV was given to patients for severe pain when VAS was ≥5. The total dose of additional analgesic (inj. tramadol) was also recorded.

Statistical analysis

Analysis was done with the help of IBM SPSS statistics version 26 (IBM Corp., Armonk, NY). Descriptive statistics of the variable from the data collected were carried out. Different parameters were compared using the chi-squared test (data like sex of the patients, ASA grade, postoperative nausea vomiting, etc.) and the unpaired t-test (data like VAS score, hemodynamic parameters, the total dose of tramadol used, etc.). P-value <0.05 was considered significant.

## Results

Table 2 shows the demographic data of patients. There was no statistically significant difference among the groups in terms of demographic variables.

**Table 2:** Demographic profile of the patients ASA: American Society of Anaesthesia

Variable	Group 1 (n=30)	Group 2 (n=30)	P-value
Age	40.9±11.7 (Mean±SD)	36.9±12.4 (Mean±SD)	7.3
Sex			0.9
Male	0	8 (27%)	
Female	30 (100%)	22 (73.3%)	
ASA			0.2
1	14 (46.7%)	12 (40%)	
2	16 (53.3%)	18 (60%)	

Table 3 shows the mean VAS score for abdominal pain at rest (abdominal muscle not used for cough or anything else) in all postoperative periods. At the 0 postoperative hour, the mean VAS score for abdominal pain in groups 1 and 2 was statistically not significant (p=0.12). At the first postoperative hour, the mean VAS score was statistically significant (p=0.009). At the second postoperative hour, the mean VAS score was statistically significant (p=0.038). At the fourth postoperative hour, the mean VAS score was statistically significant (p=0.00). At the eighth postoperative hour, the mean VAS score was statistically significant (p=0.0). Similarly, at the twelfth hour and twenty-fourth postoperative hour, the VAS score was significantly low in group 1. So from the first postoperative hour till the twenty-fourth hour, group 1 showed a significantly lower VAS score.

**Table 3:** Mean VAS score for abdominal pain at all postoperative hours VAS: visual analog scale

Time (in Hours)	Group 1 (Mean VAS Score)	Group 2 (Mean VAS Score)	P-value
0	2.93	3.33	0.12
1	3.13	3.73	0.009
2	2.9	3.3	0.038
4	2.2	2.93	0.00
8	1.6	2.23	0.00
12	1.07	1.43	0.007
24	0.77	0.86	0.002

Table 4 shows the mean artery pressure (MAP), heart rate (HR), and SPO2 in all postoperative periods. The mean MAP of both groups at various time intervals in the postoperative period was not statistically significant at all time intervals (p˃0.05). The mean HR of both the groups at various time intervals in the postoperative period was statistically not significant at all time intervals (p˃0.05). The mean SPO2 of both the groups at various time intervals in the postoperative period was not statistically significant at all time intervals (p˃0.05).

**Table 4:** Hemodynamics in postoperative hours

Vitals	Time (in Hrs)	Group 1	Group 2	P-value
MEAN ARTERIAL PRESSURE (MAP)	0	99.64	99.83	0.9
	1	98.92	98.49	0.7
	2	95.09	96.18	0.6
	4	93.04	94.14	0.5
	8	93.61	90.69	0.1
	12	90.91	91.67	0.7
	24	90.13	93.18	0.1
	0	79.90	80.73	0.7
MEAN HEART RATE (MHR)	1	78.83	77.80	0.7
	2	83.87	77.70	0.06
	4	87.37	85.10	0.3
	8	83.43	82.13	0.5
	12	85.83	83.47	0.3
	24	78.97	78.50	0.7
MEAN SPO2 (OXYGEN SATURATION)	0	98.63	99.03	0.3
	1	99.80	99.87	0.5
	2	99.83	99.93	0.3
	4	99.73	99.90	0.2
	8	99.87	99.80	0.6
	12	99.83	99.90	0.5
	24	99.93	99.93	1.0

Table 5 shows the number of patients who had postoperative nausea and vomiting (PONV). A total of 19 patients complained of PONV. In group 1, eight patients experienced PONV and in group 2, 11 patients experienced PONV. No statistically significant difference was found among the groups in terms of frequency of nausea and vomiting as p=0.4 (˃0.05).

**Table 5:** Adverse effects (postoperative nausea vomiting)

PONV (Postoperative Nausea and Vomiting)	Group 1 (n=30)	Group 2 (n=30)	P-value
Present	8	11	0.4
Absent	22	19	
Total	30 (n=30)	30 (n=30)	

Table 6 shows the total dose of tramadol used. As diclofenac was given to all patients routinely postoperatively, both the groups received a total of 150 mg in 24 hours. But tramadol consumption was higher in group 2 than group 1, and it was statistically significant, as p=0.003 (<0.05).

**Table 6:** Postoperative analgesic use (total dose of tramadol used)

GROUPS	Mean total dose (mg)	P-value
Group 1	23.33	0.003
Group 2	60.00	

## Discussion

Pain after laparoscopic cholecystectomy (LC) is very common, so multimodal pain management after surgery is very important to make this surgery more successful. Hence, we evaluated postoperative pain by comparing the effect of intraperitoneal and port-site infiltration of bupivacaine for pain relief following LC.

Demographic data

Age

In our study, patients of age 20-70 years were included. Age below 20 years and above 70 years were excluded from the study, as they represent a different subset of the population. The mean age of the patients in our study was 40.9±11.7 in group 1 and 36.9±12.4 in group 2. These data are almost as similar to that in the Gupta RS et al. study, where the mean age was 38.7±8.3 (group A), 39.4±10 (group B), and 41.05±11.6 (group C) [10].

Sex

In our study, out of 60 patients, 52 (87%) were female and eight (13%) were male. It is consistent with available literature suggestive of increased incidence of gallstones in females.

ASA Grade

We included patients of ASA grades 1 and 2. In our study, a total of 26 patients were from ASA grade 1 and 34 patients were from ASA grade 2. The ratio of ASA 1 and ASA 2 in group 1 was 7:8 and in group 2, it was 6:9. These data are supported by Altuntas et al. [11].

Postoperative pain data

The intensity of pain was demonstrated by the VAS. Pain from port sites was felt more than the visceral type of pain during the first 24 hours of surgery. Port-site infiltration of LA (0.5% bupivacaine) reduced the immediate parietal pain significantly and helped reduce the requirement of postoperative analgesics. and patients who received the intraperitoneal instillation of LA (0.5% bupivacaine) intraoperatively required a significantly higher dose of analgesia. Our study shows differences in mean VAS scores between the pre-incisional infiltration group and the intraperitoneal instillation group from the first to twelfth postoperative hours. Mean VAS scores at maximum postoperative hours were found to be statistically significant for the group. VAS scores and tramadol requirements were lower in patients who underwent local anesthetic infiltration in the trocar insertion sites than those treated with the intraperitoneal instillation of LA. Total tramadol requirement was lower in patients in whom 0.5% bupivacaine was administered in the trocar incision site than patients in whom intraperitoneal 0.5% bupivacaine was administered. The results are similar with Gouda M et al. [12], who found intra-incisional infiltration with levobupivacaine is better than intraperitoneal instillation to reduce early postoperative pain after LC. Altuntas et al. demonstrated that LA infiltration over trocar sites was more effective for postoperative analgesia [11]. Pandove PK et al. also concluded from their study that the infiltration of 0.25% bupivacaine at all trocar sites with or without infiltration in the gall bladder fossa is an effective method of postoperative pain relief when compared to the infiltration of gall bladder fossa alone [13].

Postoperative hemodynamics data

Vital parameters like MAP, HR, and SPO2 are important indicators of patients' comfort. In this study, due to adequate pain relief, all patients were hemodynamically stable in the postoperative period. No significant difference was seen between the two groups for MAP, HR, and SPO2. Similar results were found in the study conducted by Altuntas et al. [11].

Adverse effects data

Postoperative side effects were also not many in patients because of adequate pain relief and less requirement of analgesics. Only nausea and, rarely, vomiting were found in some patients. No other adverse effect was seen. In this study, PONV was found in eight (27%) patients in the port site LA infiltration group and in 11 (37%) patients in the intraperitoneal LA instillation group. This difference was not statistically significant. In this study, the total dose of bupivacaine was kept well below the toxic dose, so any kinds of side effects were not observed because of the LA administration. This result is similar to Altuntas et al. [11].

Postoperative analgesic use data

In this study, a significant difference in postoperative analgesic consumption (tramadol) was observed between the two groups. A larger number of patients required additional analgesia in the intraperitoneal instillation group as compared to the pre-incisional infiltration group. Also, the mean dose of tramadol used was significantly higher in the intraperitoneal instillation group. This suggests that intraperitoneal LA administration is partially effective, and LA infiltration in the port sites is more effective than intraperitoneal LA administration. This result is similar to the study by Altuntas et al. [11], where the total dose of morphine required was 1.5 times less in the trocar site LA infiltration group than the intraperitoneal LA group and was two times less than the control group. The results are also consistent with the findings of Gupta RS et al. [10], Pasqualucci [6], and Bisgaard [14].

The limitation of this study was that a drain was kept in order to identify possible bile leakages in many cases. Drain application may have caused a loss of local anesthetic solution and decreased the analgesic efficacy in patients who received intraperitoneal local anesthetic instillation.

## Conclusions

It can be concluded that pre-incisional LA infiltration in port sites is an easy, affordable, reliable, and effective method that provides good analgesia in the immediate postoperative period after LC.
